# What's on YOUR Facebook profile? Evaluation of an educational intervention to promote appropriate use of privacy settings by medical students on social networking sites

**DOI:** 10.3402/meo.v20.28708

**Published:** 2015-07-20

**Authors:** Jennifer M. Walton, Jonathan White, Shelley Ross

**Affiliations:** 1 Department of Pediatrics, Faculty of Medicine & Dentistry, University of Alberta, Edmonton, AB, Canada; 2 Department of Surgery, Faculty of Medicine & Dentistry, University of Alberta, Edmonton, AB, Canada; 3 Department of Family Medicine, Faculty of Medicine & Dentistry, University of Alberta, Edmonton, AB, Canada

**Keywords:** social media, professionalism, medical education

## Abstract

**Background:**

The rise of social media has led to growing concerns about the potential implications of ‘unprofessional’ postings by physicians and medical students on individuals, institutions, and the medical profession. Relevant and effective guidelines have been difficult to develop and enforce, and there is a need for students and physicians to consider how their online activities may be perceived in the context of their professional roles. The purpose of this project was to examine the Internet presence of a graduating Canadian medical school class by scanning students’ public profiles on the social media site Facebook, incorporate this information into an educational activity addressing professionalism and social media, and evaluate the impact of this activity on student behavior.

**Methods:**

A systematic search for public Facebook profiles of each member of the class was conducted, and data were collected on the types of publicly visible material. These were presented as part of an educational session on social media and professionalism. One month later, the Facebook search was repeated.

**Results:**

Of 152 students in the class, profiles were found for 121 (79.8%). The majority of students used appropriately restrictive privacy settings; however, a significant minority had publicly visible information, including comments, photographs, location, and status as a medical student. The educational innovation was well received with more than 90% of students agreeing that this topic was important and well addressed. A follow-up search found that many students had altered their privacy settings to make less information publicly available.

**Conclusions:**

A small but significant proportion of students share potentially unprofessional content on social media. An interactive educational intervention, which includes specific disclosure of how participants appear to others on social media, resulted in a significant change in student behavior.

In recent years, the use of social networking sites such as Facebook has become nearly ubiquitous among Canadian post-secondary students. As of January 2013, more than 18 million Canadians were Facebook users, which comprises 65% of the online population (1). With the burgeoning use of sites such as Facebook and Twitter, individuals are able to easily share a great deal of information with a wide audience, including personal details which previously would be shared only within a circle of closer acquaintances. Social media is playing an increasing role in medical education; a recent meta-analysis (2) details a number of potential benefits of further integration of social media into education, including improved learner engagement, collaboration, and feedback. Several studies have attempted to quantify medical student and resident use of social media sites such as Facebook. Voluntary anonymous surveys have found that between 75 and 96% of students and residents self-report as active Facebook users (3–5). A systematic search of Facebook for all medical students and residents at one American medical school in 2007 found that 45% of medical trainees had a Facebook account (6), and a follow-up study at the same institution 2 years later found that this had increased to 40% of residents and 70% of medical students (7). Importantly, these types of studies likely underestimate the prevalence of social media use, as it is possible to ‘hide’ one's account using the platform's privacy settings or by simply setting up the account under a different name.

## Potential implications of social media use on professionalism

With the rising use of social media by health care students and practitioners, there are increasing concerns about potential professionalism implications, particularly related to breaches of patient confidentiality, professional boundaries, and depiction of ‘unprofessional’ behaviors. Although the body of research on the use of social media by students, residents, and practicing physicians is relatively new, these concerns seem well founded. A survey of US medical schools found that 60% of deans had dealt with incidents related to students’ posting of unprofessional content on the Internet. Although overt breaches of patient confidentiality were relatively unusual, posting of inappropriate material such as profane or derogatory language, sexually suggestive material, and depictions of substance use or intoxication were more common (8). An observational study of medical students’ profiles at a US medical school found that more than half of students’ profiles contained photographs depicting alcohol consumption, and 30% displayed professionally inappropriate content such as drunkenness, sexually suggestive attire, and potential violations of patient privacy, particularly in international settings (6). A small study of Taiwanese medical students suggested that increased self-reported use of social media sites such as Facebook was correlated with lower scores on a self-administered medical professionalism scale (9). Finally, practicing physicians are not immune from these issues; a survey of medical boards in the United States found that 57% had at least one instance of unprofessional online activity resulting in serious disciplinary action such as license suspension, restriction, or revocation (10).

## Defining online professionalism

Survey data suggest that most medical students report taking steps to limit the amount of information publicly available on social media profiles (5); however, there is no clear consensus about what constitutes appropriate or ‘professional’ in this realm. A recent large-scale survey study found that students reported divergent opinions about online professionalism in the non-academic use of social media: whereas some students believed that maintaining a professional image should not be context specific, many respondents felt that they should not be held to higher standards than the general public (11). In another study, analysis of data from focus groups of students found that there was general agreement that clear breaches of patient confidentiality are inappropriate, but there was little other common ground about appropriate online behavior (12). Several recent studies have begun to explore the perspectives of the public on professionalism and social media. One such study asked members of the public, physician faculty members, and medical students to rate mock Facebook profiles of medical students depicting a range of behaviors. Overall, faculty and public perceptions were similarly conservative, whereas medical students had a more liberal view of material considered appropriate for sharing, and were more likely to post material that medical school faculty and the public would consider unprofessional (13). Another study asked 250 participants to rate a fictional physician's professionalism based on a Facebook profile with only professional content, personal content depicting healthy behaviors, or personal content depicting unhealthy behaviors. Physicians were deemed most professional when their profile depicted healthy personal and least professional when it contained examples of unhealthy behaviors (14).

## Guidelines on social media use

With increased awareness of the potential impacts of social media postings by medical students and practicing physicians, there are growing calls for the development of guidelines and policies to delineate appropriate behaviors. However, the rapid uptake and constant evolution of these technologies result in a ‘moving target’, making it difficult for academic institutions and regulatory bodies to create appropriate policies that remain relevant to the changing landscape. Organizations such as the Canadian Medical Association (15) and the Canadian Federation of Medical Students (16) have recently begun to formally address this issue by releasing official positions and guidelines. However, these are often necessarily vague and defer to the practitioner's personal judgment in specific instances. Given the lack of consensus about what constitutes professional behavior, and the demonstrated disconnect between what medical students have been known to post and what the profession and the public considers appropriate, there is a need for medical school curricula to specifically address professionalism in the context of social media.

## Educating students about professionalism and social media

To date, much of the published literature on social media in medical education has related to either how social media can be used in educationally innovative ways (2) or cautionary studies outlining the myriad of ways professionalism may be impacted by social media (8, 10, 17). Despite increased awareness of the need to formally address professional implications of social media within medical school curricula, there remains very little literature on specific educational strategies. One recent manuscript (18) described the integration of social media into a professionalism course using didactic lecture and small group discussions, and found that the session was generally well received and many students expressed motivation to monitor and edit their social media presence more carefully, although there was no attempt to determine whether students actually changed their behavior.

The objectives of this exploratory pre–post study were to 1) examine the presence of a graduating class of Canadian medical students on a popular social networking site (Facebook) and 2) develop and then evaluate the impact of an educational intervention focused on professionalism and social media on student behavior.

## Methods

This study took place at a Canadian medical school during the 2011/2012 academic year. This institution has a traditional 4-year graduate entry MD program, with 2 years of preclinical instruction and 2 years of clinical experience. All members of the graduating class were included in both the data collection and educational intervention components. The study was reviewed and approved by the institutional research ethics board. Given that this study occurred in the context of evaluation of an educational activity, the public nature of the data accessed, and the minimal risks to participants, written informed consent of participants was not required. During the data collection period, the faculty member who viewed student profiles was not involved in any formal assessments of students in the class.

### Data collection

Prior to the educational activity, the social networking site Facebook was systematically searched for profiles for each member of the graduating class. In order to replicate what a member of the public with a Facebook account would find if they looked for a given student, the search was performed from the account of a faculty member who was not a ‘friend’ or ‘friend of a friend’ of any member of the class. A multistep search strategy was used. Initially, a simple name search was used, filtering by city and/or university where multiple individuals with the same name were found. Although the privacy settings of Facebook at the time allowed an individual to make himself ‘unsearchable’, a profile could still be located by searching within the ‘friends list’ of individuals in an individual's social network. Therefore, if a student was not found through a simple name search, a secondary search of the friends’ lists of several randomly selected classmates was performed. This process was continued until no new profiles were found in the ‘friends’ lists of two consecutive class members.

Once a profile was located, data were collected on the type of personal information visible (including whether individuals self-identified as medical students), characteristics of the profile picture, types of personal photographs viewable, and the nature of postings such as status updates, comments on other friends’ profiles, and links to other pages or websites.

### Educational intervention

Following the data collection period, students attended a mandatory whole-class educational session on the topic of social media in medicine. This newly developed session was part of a longitudinal course on practical aspects of professionalism and was mandatory for all students. Given the paucity of evidence-based educational strategies in this domain, content was developed based on consensus of local faculty and consultation with key stakeholders including those involved with addressing issues of student professionalism. The session was 3 h long, and included both didactic and interactive elements. Material covered included key definitions, opportunities and risks of social media in health care, and a general summary of positions of various professional organizations and regulatory bodies. Several faculty members presented their own approaches to professionalism challenges raised by Facebook. The data collected on that specific class's presence on Facebook were presented, and students were shown a demonstration of how to check which material was publicly visible on their profiles. Students were then broken into small groups for faculty-facilitated, scenario-based small group discussions. Topics of discussion included participation in online discussion forums, patient information on personal portable electronic devices, text messaging, and blogging. Finally, all students were required to submit a brief reflection on their own experiences of professionalism challenges related to social media. Given the potential for disclosure of sensitive personal information, written student reflections were not analyzed as part of this project.

### Student feedback

A three-item Likert-scale questionnaire was distributed and collected immediately following the session. Questions related to how useful students felt the material was for 1) their medical education, 2) their future medical career, and 3) the overall quality of the presentation. Narrative comments were also invited.

### Follow-up

One month following the session, the same search strategy and data collection procedure as described above were performed to determine whether students had changed their privacy settings following the educational session.

### Statistical analysis

Data were compiled and analyzed using standard descriptive measures and non-parametric comparative statistics (chi-square) in Office Excel 2003 (Microsoft Corp., Redmond, WA, USA). McNemar's test (19) was used to assess the significance of the differences between proportions of students with specific profile content available before and after the educational intervention. These analyses were conducted using the online statistics site VassarStats (20). A *p*-value of 0.05 was used to determine statistical significance in all cases.

## Results

### Demographics

At the time of this intervention, there were 152 students in the final-year class, with a ratio of male:female students of 55:45.

### Social media profiles

A search for Facebook profiles was conducted for all 152 members of the class, and profiles were found for 121 students (79.6%). Profiles for 56 students (36.8%) were found using a simple name and university search. A further 64 profiles (42.1%) were found by searching the ‘friends’ lists of four of these students. No additional profiles were found after searching ‘friends’ lists of the fifth and sixth randomly selected class members, so the search was stopped. Of note, 25 students (16.4%) were found to be using a pseudonym on their profiles. Various strategies were used to disguise proper names, including alternate spellings, transposed letters, nicknames, names spelled backwards, and middle names used as surnames. Despite these strategies, students were easy to identify from the ‘friend’ lists of classmates, particularly as many students had recognizable profile pictures. Profiles of male and female students were found with equal frequency (Pearson chi-square=0.74, *P=*0.39).

### Availability of personal information

Overall, more than half of students whose profiles were found used a recognizable photograph as a publicly visible profile picture (69/121, 57.5%). One student's profile picture was unrecognizable, however contained potentially objectionable material (profanity). Female class members were significantly more likely to use a recognizable picture of themselves than were males (Pearson chi-square=5.8, *P=*0.02).

The majority (67.7%, 81/121) of students had blocked public access to personal information such as current location, birth date, and institutional affiliations. Another 25% (30/121) had blocked sensitive information but still disclosed less personal details such as hometown, former employers, etc. More than half of students (66/121) allowed their lists of friends to be publicly visible. Six students (5%) identified themselves specifically as medical students at this institution. One student's information included a quotation that contained mild profanity.

### Visible comments and posts

As Facebook users post photographs, video, comments, status updates, links, and other information, this activity is summarized on their profile pages. Unless intentionally disabled, any friends of the user are also able to post comments and other material to an individual's profile. In this sample of students, 24.7% (30/121) had completely blocked this posted material from public view, whereas 70.2% (85/121) had blocked most content aside from occasional comments and notifications of when they had been added to another individual's list of friends. Six students (5%) had all content publicly visible, and two of these students had frequent references to medicine and/or medical school. No overt breaches of patient confidentiality were observed on these publicly visible pages.

### Publicly visible photographs

One of the frequently used features of Facebook is the ability to post photographs for others to view. Users are able to adjust privacy settings to limit who can see their photographs. For the profiles found in this study, 70.2% (85/121) of students had blocked public access to all of their photographs. Several others (8/121, 6.6%) had a few old profile pictures available, but no others. However, there were 28 students (23%) who had large numbers of publicly accessible photographs. A number of these students (11/28) had photographs of members of the medical school class in both academic (workshops, conferences) and social (parties, sports, games etc.) contexts. Six students had photos with questionable content including overt alcohol use and individuals in sexually suggestive poses or attire. There were no publicly viewable photographs of patients or information that might be perceived as a threat to patient confidentiality.

### Student evaluation of educational session

Completed evaluations were received from 80% of the class. Overall, student perceptions of the educational intervention were very positive. On a 5-point Likert scale, the mean responses to the questions ‘This topic is important for my medical education’, ‘This topic is important for my medical career’, and ‘This topic was addressed in an effective session’ were 4.3, 4.4, and 4.4 out of 5, respectively. Narrative comments commonly reflected an appreciation that this topic was being formally addressed, interest in faculty approaches to social media, and surprise at how much personal information could be found.

### Follow-up

A repeat search for all class members one month following the educational intervention revealed that many students had changed their privacy settings to further restrict public access to information on their Facebook accounts (Table 1). Fewer overall students could be found by any search strategy, and in particular, there was a significant decrease in the proportion that could be found using only a simple name search. Significantly, fewer students displayed personal information or friends lists. Finally, there was a significant reduction in the number of students who openly displayed large numbers of personal photographs; only two students continued to publicly post photographs directly related to medical school, and only three continued to display photographs depicting alcohol consumption or inappropriate attire. There were no significant changes in the proportions of students who displayed their medical student status, recognizable profile pictures, or provided open access to comments and postings.

**Table 1:** Information visible on students’ Facebook profiles before and after educational intervention *N=*152

	Pre (%)	Post (%)	*P* a
Profile found by any means	79.6	75.6	0.04
Profile found by simple name search	36.8	28.9	0.008
Friends’ list visible	43.4	32.2	0.000015
Profile picture recognizable	59.1	55.6	NS
Medical student status mentioned	4.0	1.9	NS
Personal information disclosed	33.1	24.5	0.000012
Comments and other activity visible	4.0	4.0	NS
Many personal photographs visible	18.4	9.8	0.0002
Any ‘questionable’ content	6.6	2.0	0.04

a McNemar's test of significance between two correlated proportions. *P*<0.05 used to determine statistical significance.

## Discussion

This is the first study to systematically examine the online social media presence of a single class of medical students and to use this information in an educational context to encourage students to reflect on the online image that they portray.

Our ability to find a Facebook profile for 80% of members of a class of graduating medical students was similar to the findings of similar searches for Facebook profiles of veterinary medicine (21) and first-year pharmacy students (22), where profiles were found for 73 and 77%, respectively. Although we found that most medical students already used appropriately strict privacy settings to limit public access to their personal information prior to the educational intervention, a significant minority of students allowed public access to personal information. Of greatest concern were the nearly 25% of students who had comments and multiple photos publicly available, particularly those whose photos depicted behavior that others might consider ‘unprofessional’. This is in contradiction to the findings of a survey where the majority of senior medical students reported that their social media profiles were completely private (23), suggesting that students may not actually be aware of how much of the information on their social media profiles is visible to the public. This is supported by student comments in the evaluation of our session in which many reported surprise and alarm about how much of their personal information was publicly available.

Inappropriate posts on social media may have consequences to individual students as well as to institutions and professions. For example, screening social media profiles as part of the residency selection process is an area of increasing interest and debate. Exploration of the social media presence of applicants to orthopedic surgery (24) and otolaryngology (25) residency programs located Facebook profiles for 46–51% of applicants. Both studies found that 85% of candidates publicly shared more than basic demographic information, and 11–16% contained ‘unprofessional’ content. Not surprisingly, a growing number of residency program directors are screening candidates’ social media profiles (26) and many report down-ranking candidates based on unprofessional content. Mainstream media interest in the issue of inappropriate social media behavior of students in professional programs has been piqued by recent incidents such as one at a Canadian dental school (27), and stories such as this are capturing the attention of the public as well as regulatory bodies (28). There is a very real risk that unprofessional behavior on social media by a minority of individuals may have a negative impact on the already fragile public trust in professions such as medicine (14).

A recent commentary conceptualized medical education on social media professionalism in terms of a ‘hierarchy of needs’, with the most basic need being safety (knowing which behaviors could compromise one's career), advancing to reflection on one's social media image, and culminating in discovery, or using social media to innovate and improve health outcomes (29). To date, most reports on educational initiatives have been focused at the level of safety by reminding students of the ‘rules’ and potential harms of social media use. For example, one such study examined pharmacy students’ Facebook profiles before and after email dissemination of a social media policy (22). Although they did demonstrate a significant decrease in the number of students with publicly available posts, they did not find any changes in the proportions of students posting personal information, friends’ lists, or photographs following the intervention. In contrast, our intervention, which was aimed at encouraging students to consider the perceptions of others who might view their online activities, and was thus targeted more at the level of reflection, was associated with significant decreases in many types of publicly visible material.

There are a number of limitations of this study. It reflects data from a single class at a single medical school, and may not be representative of other students or other institutions, although there is no reason to believe that students at this institution are any different than others with respect to their use of social media. Furthermore, it is impossible to determine whether the behavior change we observed was due to the education session as a whole, or simply a result of the students becoming aware that a faculty member had examined their online profiles. Anecdotally however, when this session has been repeated in subsequent years, searches of student profiles have unearthed similar results, despite students hearing from previous classes that their profiles could be searched. This suggests that the behavior change was more likely an impact of knowing that one's own profile had been examined, rather than a more abstract sense that student profiles could be examined. We examined only publicly visible elements of each student's Facebook profile; therefore, it is not possible to draw any conclusions about student behavior on pages protected by privacy settings. It is very likely that there are many more instances of material that could be construed as ‘unprofessional’ that are hidden from public view. Finally, Facebook is only one of the many social media platforms commonly used by students, and it is unclear whether similar strategies could be used to address issues related to blogging, Twitter, YouTube, etc.

There were no significant problems implementing the innovation: it was introduced during an existing course that was transitioning its focus to professionalism in the clinical context. It was identified as a priority topic by the faculty, so finding curricular time was not an issue. Searching Facebook for profiles of every student did prove to be a time-consuming process; however, it provided a novel ‘hook’ to grab students’ attention and made the session much more memorable. Future studies could address whether there are differences in behavior change for sessions which present class-specific versus generic data.

Finally, it is important to situate this study in the context of ongoing evolution of the concepts of professionalism. It is unclear whether problematic social media activity is in fact a new variety of ‘unprofessionalism’ or simply reflects behaviors that have always existed but are now magnified by the perceived anonymity and wide reach of the Internet. There is a fine line between encouraging students to limit access to personal aspects of their online activities to those they know and trust, and over-focusing on the dangers of self-expression and thus prompting suppression of authentic behavior.

## Conclusions

Even within a cohort of senior medical students who are very familiar with technology, many individuals are not as vigilant about their privacy settings on social media sites as one might hope. Reflecting this information back to them as part of an educational intervention proved to be engaging, popular, and ultimately a highly effective strategy for both opening a dialogue and changing student behavior.
